# Exercise for Hepatic Fat Accumulation in Type 2 Diabetic Subjects

**DOI:** 10.1155/2013/309191

**Published:** 2013-09-01

**Authors:** Elisabetta Bacchi, Paolo Moghetti

**Affiliations:** Unit of Endocrinology, Diabetes and Metabolism, Department of Medicine, University and Azienda Ospedaliera Universitaria Integrata of Verona, Piazzale Stefani 1, 37126 Verona, Italy

## Abstract

Type 2 diabetes is characterized by frequent ectopic fat accumulation in several tissues and organs. In particular, a number of studies showed that these subjects frequently have hepatic fat accumulation, which may play a role in the metabolic abnormalities typical of diabetes and has been also linked to increased risk for cardiovascular disease. In the last decade, the effect of exercise on ectopic fat content of type 2 diabetic patients has raised growing interest. However, there are only a few small randomized controlled trials on this topic. Results from these intervention studies indicate that exercise training, independent of dietary modifications, may reduce hepatic fat content and serum transaminases in these patients, suggesting that exercise *per se* may be an effective strategy to be combined with the traditional dietary interventions. As regards the different training modalities, there is recent evidence that both aerobic and resistance exercise may equally reduce hepatic fat accumulation in type 2 diabetic subjects. However, information regarding the effect of exercise on liver histology and fat accumulation in other ectopic sites is still very limited.

## 1. Introduction

Type 2 diabetes is characterized by frequent ectopic fat accumulation in several tissues and organs (liver, skeletal muscle, heart, and pancreas) and unfavorable adipose tissue distribution [1]. Ectopic fat accumulation is defined by the deposition of triglycerides within cells of nonadipose tissues, which normally contain only small amounts of fat. Over the past decade, magnetic resonance (MR) spectroscopy has been used as the gold standard technique for noninvasive quantification of intramyocellular, hepatic, and more recently myocardial and pancreatic lipids. However, ectopic fat content can also be assessed by other methods, such as computed tomography and magnetic resonance imaging, which provide data on hepatic fat content that correlate very well with those obtained by the proton MR spectroscopy as well as with the histopathologic findings [2].

In particular, a number of studies have shown that subjects with type 2 diabetes frequently suffer from nonalcoholic fatty liver disease (NAFLD), a term which includes a variety of pathological conditions, from simple steatosis to nonalcoholic steatohepatitis and cirrhosis. Available data suggest that approximately 50% to 70% of patients with type 2 diabetes have NAFLD, whereas the frequency of ectopic fat infiltration in other tissues has yet to be fully investigated. Interestingly, this phenomenon seems to have prominent clinical implications. In particular, there is evidence that NAFLD may play a role in the progression of insulin resistance and in several other metabolic abnormalities typical of diabetes, as well as in the increased risk for cardiovascular disease of these subjects [3].

The mechanisms underlying the relationships between type 2 diabetes and ectopic fat accumulation in the body are still largely unclear, and the specific approach to this phenomenon has yet to be determined. However, it is widely accepted that lifestyle changes, that is, diet and exercise, are the mainstay of treatment, as they may improve metabolic control and reduce body fat in these patients. Conversely, limited information is available on the effects of pharmacological treatments on ectopic fat infiltration.

In this paper we review the literature regarding the role of exercise on ectopic fat accumulation in subjects with type 2 diabetes. It should be noted that most studies on this issue specifically analyzed NAFLD. 

## 2. Strategies in the Treatment of Ectopic Fat Accumulation

According to the present knowledge about pathophysiology of ectopic fat accumulation, there are several potential targets for this phenomenon [4]. Weight loss in overweight/obese patients is an obvious target and the widely accepted milestone of treatment. In a recent meta-analysis or randomized trials, it was observed that a weight loss of at least 7% is effective in improving histological disease activity, although it was achieved by less than 50% of patients [5]. Additional proposed strategies include targeting insulin resistance, hyperglycemia, dyslipidemia, oxidative stress, and inflammation. Research focusing on specific dietary components suggests that both macronutrients and micronutrients may play a role in the development of NAFLD [6]. From this point of view, there is evidence of adverse effects of fructose and favourable effects of polyunsaturated fatty acids (PUFA), possibly linked to opposite effects of these nutrients on inflammation. The role of vitamins and minerals in this field is also under investigation. Interestingly, lifestyle improvement may favourably affect all these potential mechanisms underlying ectopic fat accumulation.

Among the pharmacological options, statins showed some efficacy in the treatment of NAFLD [5]. As regards antidiabetic medications, insulin sensitizers and glucagon-like peptide-1 (GLP-1) analogs appear to be the most interesting options. However, the literature on this topic is still limited. Several studies assessed the potential efficacy on NAFLD of the insulin sensitizer metformin, which is the first-line medication in the treatment of type 2 diabetes and primarily improves hepatic insulin sensitivity. These studies consistently showed that metformin may reduce serum levels of liver enzymes, a surrogate marker of NAFLD. However, most of these studies were uncontrolled and liver histology results were inconsistent [4, 5].

The effect on NAFLD of thiazolidinediones, another class of insulin sensitizers, which are agonists of peroxisome proliferator activated gamma (PPAR-gamma) receptor, was also investigated by several studies. These drugs improve insulin action primarily at the skeletal muscle level. However, PPAR-gamma receptor is highly expressed in adipose tissue. Moreover, these medications induce a redistribution of body fat depots, making these molecules of particular interest for targeting ectopic fat accumulation. Available data from a few randomized trials support this hypothesis, showing that thiazolidinediones may improve liver histology [5]. In particular, these drugs consistently reduced hepatic steatosis and inflammation; in addition, in patients with stable stage fibrosis, they significantly reduced progression of fibrosis [5]. Nonetheless, large RCTs are needed before we can consider thiazolidinediones a specific treatment for ectopic fat accumulation.

As regards GLP-1 analogs, which belong to the incretin class and favour weight loss, there are some ongoing randomized trials. Preliminary data have shown the reduction of liver enzyme levels and hepatic steatosis after treatment with these drugs, suggesting they could be potentially useful in targeting NAFLD in diabetic patients.

## 3. The Role of Exercise Training in Type 2 Diabetes

Although increased physical activity has for decades been considered a first-line issue in the treatment of type 2 diabetes, the role of exercise training in these subjects has recently raised renewed and considerable interest in both clinical and scientific terms. In this regard, a recent joint position statement of the American College of Sport Medicine and the American Diabetes Association suggested that, whenever possible, both aerobic and resistance exercise training for subjects with type 2 diabetes, should be used to improve glycemic control, cardiovascular risk factors, and body composition [7]. Aerobic and resistance exercise training share some general effects but differ in their specific characteristics. Aerobic training, such as walking or cycling, involves repetitive and rhythmic contraction of large muscle groups, promoting cardiorespiratory fitness. Conversely, resistance training, such as weightlifting, typically engages relatively slow, high force contractions, promoting musculoskeletal fitness and stimulating the increase in muscle proteins and muscle cross-sectional area.

In patients with type 2 diabetes, some recent head-to-head RCTs have shown that both aerobic and resistance training may elicit similar results in terms of a number of endpoints, such as glucose control, insulin sensitivity, and body composition [8–10]. Although the results of some of these studies suggested that combination training could be more effective in these subjects than aerobic or resistance training alone [9, 10], it should be pointed out that in these trials exercise volume was higher in the combination groups, precluding a definitive answer to this crucial question.

As regards ectopic fat, regular physical activity may reduce its content through several different mechanisms, including increased hepatic and muscle fatty acid oxidation, reduced postprandial hepatic lipogenesis, and reduced fatty acid and proinflammatory molecule flow to the liver and other organs. However, to date only a few intervention studies have assessed the effect of exercise, either alone or in combination with diet, on ectopic (especially hepatic) fat content [11–16] (Table 1). Moreover, most of these studies included a combination of exercise and caloric restriction, making it difficult to assess the role of exercise *per se*.

## 4. Effect of Exercise Training on Liver Fat Content in Type 2 Diabetes

Aerobic exercise is usually recommended in the management of NAFLD. However, not all subjects are able to perform this type of exercise. Moreover, until now only one randomized controlled trial has assessed the effect of exercise alone on hepatic fat content in subjects with type 2 diabetes [16], while four RCTs have compared the effect of combined interventions with both aerobic exercise and hypocaloric diet versus diet alone or standard diabetes support and education programs [11, 13–15].

We have recently randomized 31 type 2 diabetic patients with NAFLD to regular training with either aerobic or resistance exercise. Interestingly, after 4 months hepatic fat content was markedly reduced, to a similar extent, in both groups, showing that these exercise modalities are equally effective in type 2 diabetic patients with NAFLD [16]. It is noteworthy that hepatic steatosis disappeared in about 25% of these diabetic subjects. This reduction in hepatic fat accumulation was accompanied by concurrent, mild but significant improvements in both anthropometric [BMI, total and truncal body fat, abdominal visceral (VAT), and subcutaneous (SAT) adipose tissue] and metabolic features (insulin sensitivity, HbA1c, triglycerides). These results were independent of the effect of diet, as participants were instructed to maintain their baseline calorie intake by consuming healthy self-selected foods, and compliance to dietary recommendations was confirmed by a stable body weight in the pre-intervention period and the results of the food recall at the end of intervention.

Conversely, Bonekamp et al. in a sample of 45 subjects with type 2 diabetes have assessed the effect of combined, moderate intensity training with both aerobic and resistance exercise, without any associated caloric restriction [12]. Their preliminary results suggested that combined exercise training may reduce hepatic fat content, independently of any changes in body composition and metabolic features.

In agreement with these findings, a one-month aerobic training intervention, carried out in a sample of 19 non-diabetic obese individuals, reported a 21% reduction of hepatic fat content, in the absence of any significant body weight reduction [17]. Similarly, Hallsworth et al. [18] have recently reported that in 18 subjects with NAFLD, some of them with type 2 diabetes, there was a 13% reduction of hepatic fat content after 8 weeks of resistance exercise, without any changes in body weight.

Other studies reported that, in the short term, hepatic fat content is similarly reduced after diet only or exercise *plus* diet interventions [11, 13–15]. Tamura et al. [11], in 14 type 2 diabetic patients, showed a similar decrease of hepatic fat content, with a reduction of body mass index, after two weeks of diet only (60% carbohydrate, 25% fat, 15% protein, and mean total energy intake of 27.9 kcal/kg ideal body weight) or diet *plus* exercise (30 minutes of moderate intensity aerobic exercise, 5-6 times a week). More recently, in 45 obese subjects with type 2 diabetes, Bozzetto et al. [15] compared the effect of high-carbohydrate/high fiber/low glycemic index diet or multi-unsaturated fatty acid (MUFA) diet, with or without a concurrent moderate intensity aerobic exercise program of 45 minutes 2 times a week. After 8 weeks, the authors reported that MUFA intervention, as compared with the control diet, was associated with a significant and clinically relevant reduction of hepatic fat content, without reduction in body weight. These findings were independent of the associated training program, as the reduction of liver fat content was similar in both the MUFA diet alone and the MUFA diet *plus* exercise protocols. Nonetheless, it could be hypothesized that the duration of the interventions was too short to detect differences in changes of hepatic fat content between the exercise *plus* diet group and the diet only group. Even more important, these authors reported a negligible improvement of only 1 mL/kg/min of peak oxygen uptake in the MUFA *plus* exercise group compared with the MUFA only group. Thus, it can be speculated that training volume and/or intensity were not sufficiently adequate to improve cardiorespiratory fitness of these subjects.

Two studies [13, 14] carried out in the cohort of the Look AHEAD Study—a multicenter prospective study comparing intensive lifestyle intervention, including caloric restriction and at least 175 min of moderate aerobic physical activity per week, versus standard care in diabetic subjects—reported that after 1 year of intervention there was a significant decrease in hepatic fat content and a reduced incidence of NAFLD in patients randomized to the intensive lifestyle group. In particular, the median percent decrease in steatosis was 50.8% in the intervention lifestyle group versus 22.8% in the control group [13]. In addition, 3% of participants in the intervention group versus 26% of those in the control group, who were without NAFLD at baseline, developed NAFLD during the study. Interestingly, similar changes in hepatic fat content were found in both men and women [14].

Overall, these data support the recommendation for weight loss using lifestyle changes as the first step in patients with NAFLD, including those with type 2 diabetes. In addition, they suggest that either aerobic or resistance exercise *per se* is effective in reducing hepatic fat content. Unfortunately, due to the limited information available, it remains unclear what amount/volume and what intensity of exercise is optimal in targeting a reduction of hepatic fat content.

## 5. Summary

In the last decade, the effect of exercise on ectopic, especially hepatic, fat content of type 2 diabetic patients has raised growing interest. However, there are still only a few small RCTs on this topic. Results from these intervention studies are promising, as they indicate that exercise training, independent of dietary modifications, can reduce hepatic fat content in these patients, suggesting that exercise *per se* may be an effective strategy to be combined with the traditional low calorie diet interventions.

The extent of improvement induced by exercise training in hepatic fat content appears to be quite different in these studies. These differences might be explained by differences in exercise volume and intensity, as well as in the duration of the interventions, although they can also be linked to the different techniques used for the measurement of fat content.

Future research should further address the effects of both aerobic and resistance exercise, alone or in combination, on ectopic fat accumulation of these subjects, by focusing on the differences between training protocols in terms of exercise frequency, duration, and intensity, in order to establish which training model is more effective in reducing ectopic fat accumulation and thereby counteracting the multiple adverse effects of this phenomenon.

## Figures and Tables

**Table 1:** Summary of RCTs which assessed the effect of exercise training on hepatic fat content of subjects with type 2 diabetes.

Study	Patients	Design and duration	Intervention	Technique for fat assessment	Effect of intervention		
					BMI	Body composition	Hepatic fat content
Tamura et al., (2005) [11]	14, overweight	Diet versus diet plus exercise; 2 weeks	AER exercise 50–60% VO_2max_, 30 min, 5-6 times a week; low-fat diet	H-MRS	D: −0.4 kg/m^2^ D + EX: −0.7 kg/m^2^	Body fat D: −2.4% D + EX: −2.8%	D: −2.18% D + EX: −1.48% (absolute change)

Bonekamp et al., (2008) [12]	45, obese	Exercise versus control group; 6 months	Moderate AER and weightlifting exercise, 45 min, 3 times a week; (Diet: ?)	H-MRS	No change	Body fat EX: −1.3% SAT EX: −12 cm^2^	EX: −2.5% (absolute change)

Lazo et al., (2010) [13]	96, obese	Intensive lifestyle versus standard diabetes support and education; 12 months	Moderate AER exercise, 175 min per week; moderate caloric restriction	H-MRS	−2.6 kg/m^2^	Body fat: −8.8% SAT: −6.7% VAT: −12.7%	D + EX: −50.8% DSE: −22.8% (percent change)

Albu et al., (2010) [14]	58, obese	Intensive lifestyle versus standard diabetes support and education; 12 months	Moderate AER exercise, 175 min per week; moderate caloric restriction	CT scan	D + EX: −4 kg/m^2^ in men −2.8 kg/m^2^ in women	Body fat D + EX: −27.7% in men −14% in women	D + EX: −18% in both men and women (percent change)

Bozzetto et al., (2012) [15]	45, obese	CHO/fibers versus MUFA versus CHO/fibers + exercise versus MUFA + exercise; 8 weeks	AER exercise 70% of baseline VO_2peaks_, 45 min 2 times a week	H-MRS	No change	No change	MUFA: −29% MUFA + EX: −25% (percent change)

Bacchi et al., (2013) [16]	31, overweight or obese	Aerobic versus resistance training; 4 months	AER exercise 60–65% HRR 60 min, 3 times a week; RES exercise: 9 exercises, 3 series, 10 repetitions at 70–80% 1RM, 60 min, 3 times a week; habitual diet	MRI	AER: −0.70 kg/m^2^ RES: −0.55 kg/m^2^	Body fat AER: −1.90 kg RES: −1.76 kg VAT AER: −66.8 cm^2^ RES: −38.0 cm^2^ SAT AER: −17.5 cm^2^ RES: −22.5 cm^2^	AER: −32.8% RES: −25.9% (percent change)

AER: aerobic training; CHO: carbohydrate; CT: computed tomography imaging; D: diet only; D + EX: diet plus exercise; DES: standard diabetes support and education; EX: exercise; HRR: heart rate reserve; H-MRS: proton magnetic resonance spectroscopy; MRI: magnetic resonance imaging; RES: resistance training; 1RM: one repetition maximum; SAT: subcutaneous adipose tissue; VAT: visceral adipose tissue; MUFA: multi-unsaturated fatty acid diet.
